# Molecular Cloning and Functional Analysis of the Duck *TLR4* Gene

**DOI:** 10.3390/ijms140918615

**Published:** 2013-09-10

**Authors:** Wenming Zhao, Zhengyang Huang, Yang Chen, Yang Zhang, Guanghui Rong, Chunyu Mu, Qi Xu, Guohong Chen

**Affiliations:** Jiangsu Key Laboratory for Animal Genetic, Breeding and Molecular Design, Yangzhou University, Yangzhou 225009, Jiangsu, China; E-Mails: beansgreen@outlook.com (Z.H.); youngisyang@gmail.com (Y.C.); huang137167741@yeah.net (Y.Z.); jackwong@aliyun.com (G.R.); beansgreen@sina.com (C.M.); xuqi@yzu.edu.cn (Q.X.)

**Keywords:** duck, *TLR4*, alternative splicing, expression analysis

## Abstract

Toll-like receptor 4 (TLR4) recognizes pathogen-associated molecular patterns in some animals and has been shown to be closely associated with several diseases such as tumors, atherosclerosis, and asthma. However, its function in ducks is not clear. Alternative splicing of the *TLR4* gene has been identified in pigs, sheep, mice, and other species, but has not yet been reported in the duck. In this study, alternative splicing of the duck *TLR4* gene was investigated using reverse transcription-polymerase chain reaction (RT-PCR). Duck *TLR4* gene (*duTLR4*, accession number: KF278109) was found to consist of 3367 nucleotides of coding sequence. An alternative splice form, *TLR4-b*, was identified and shown by alignment to retain the intron between exons 1 and 2. Real-time quantitative polymerase chain reaction (qPCR) analyses suggested that *duTLR4-a* (wild-type) mRNA is widely expressed in various healthy tissues, whereas *TLR4-b* is expressed at only low levels. Following stimulation of normal duck embryo fibroblasts with lipopolysaccharide, the expression of both isoforms initially increased and then decreased. Expression of the wild-type isoform subsequently increased again, while that of the variant remained low. The expression levels of wild-type *TLR4* were further analyzed by transient transfection of a pcDNA3.1(+)-*TLR4-a* overexpression vector into duck embryo fibroblasts. qRT-PCR analyses showed that after stimulation with LPS and poly(I:C) the expression levels of IL-1β, IL6, and MHC II increased with a response-efficacy relationship. Our experimental results indicate that *TLR4* plays an important role in resistance to both bacterial and viral infections in the duck.

## 1. Introduction

Toll-like receptors (TLRs) and pathogen pattern recognition receptors (PRRs) were discovered in the last century and subsequently shown to be involved in innate immunity [1]. TLRs represent a major component of the vertebrate pattern-recognition receptor system, which plays a role in detecting invading microorganisms and distinguishing between them [2,3]. To date, 13 members of the TLR family have been investigated in mammals, among which TLR4 is the most extensively studied. The various TLRs exhibit different patterns of expression, and TLR4 is most abundantly expressed in placenta and in the myelomonocytic subpopulation of leukocytes.

TLR4 recognizes the specific and highly conserved molecular patterns expressed on pathogenic microorganisms, helping to initiate and regulate the body’s immune response [4–7]. TLR4 is associated with various diseases in animals, such as tumors, atherosclerosis, and asthma. Transcript variants of *TLR4* have been found in pigs, sheep, mice, and other species, but the protein-coding potential of most variants is uncertain [8–10]. Alternative splicing of *TLR4* in the duck has not yet been reported, and its function in ducks is not clear.

In this study, we identified an alternative splicing product of the *duTLR4* gene (*TLR4-b*) but found only low expression of this variant in various healthy tissues. Next, we investigated *TLR4* expression levels using a pcDNA3.1(+)-*TLR4-a* overexpression vector that was transiently transfected into duck embryo fibroblasts. The expression of wild-type *TLR4-a* increased after stimulation with polyinosinic:polycytidylic acid [poly(I:C)]. In addition, qRT-PCR analyses showed that expression levels of interleukin *IL-1*β, *IL-6*, and major histocompatibility complex II (*MHCII*) increased after stimulation with lipopolysaccharide (LPS) or poly(I:C), revealing a response-efficacy relationship. Our experimental results confirm that TLR4 plays an important role in resistance to both bacterial and viral infections in the duck.

## 2. Results

### 2.1. Cloning and Sequence Analysis of *duTLR4*

**Figure 1:**
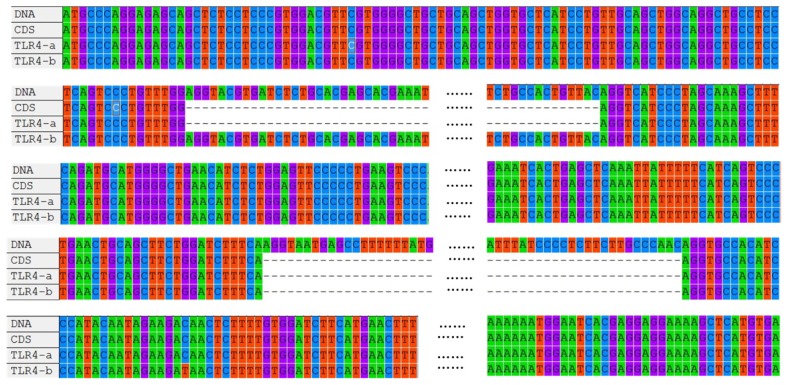
Alignment of genomic DNA, CDS, and transcript variants of the duck *TLR4* gene. “……” represents omitted bases; “DNA” represents *TLR4* DNA; “CDS” represents the *TLR4* coding sequence; “TLR4-a” represents the first transcript variant; “TLR4-b” represents the second transcript variant.

The coding sequence (CDS) of wild-type *duTLR4* (*TLR4-a*) of the Jinding duck (*Anas platyrhynchos*), determined by full-length RT-PCR and long distance (LD) PCR, was 2532 bp in length and contained three exons and two introns. Alignment of *duTLR4* mRNA (accession number: JN048668.1) and genomic DNA (accession number: JQ839148) revealed two variants (a and b) with an alternative 835 bp region between the first and second exons (Figure 1).

**Figure 2:**
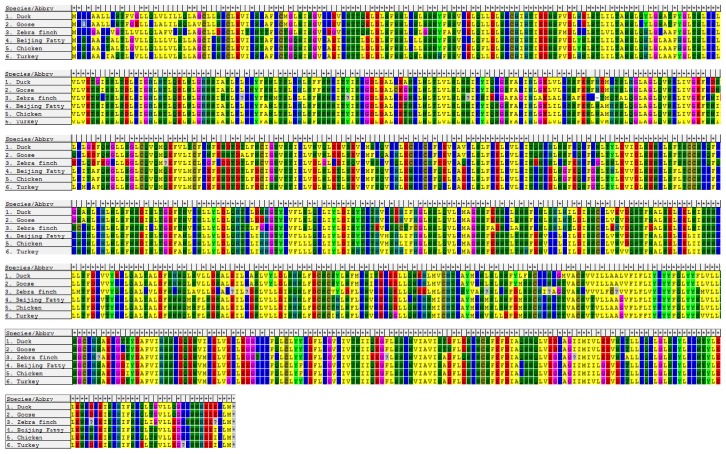
Alignment and architecture of the predicted TLR4 amino acid sequence for the Jinding duck. Amino acid sequences of TLR4 in Jinding duck (*Anas platyrhynchos*), domestic goose (*Anser* anser), Beijing Fatty (*Gallus gallus domestica*), Chicken (*Gallus gallus*), Turkey (*Meleagris gallopvo*), and Zebra finch (*Taeniopygia guttata*). Different colors represent different amino acid residue, respectively.

The entire open reading frame (ORF) of duck *TLR4* is 2532 bp (GenBank ID: JN048668.1) and encodes 833 amino acid residues (Figure 2). Not surprisingly, the nucleotide sequence of *TLR4* that we obtained is nearly identical to that previously reported for duck. The nucleotide sequence shows 96.8%, 86.5%, 86.3%, and 80.5% homology with the *TLR4* sequences of red jungle fowl, goose, turkey, zebra finch, and chicken, respectively.

**Figure 3:**
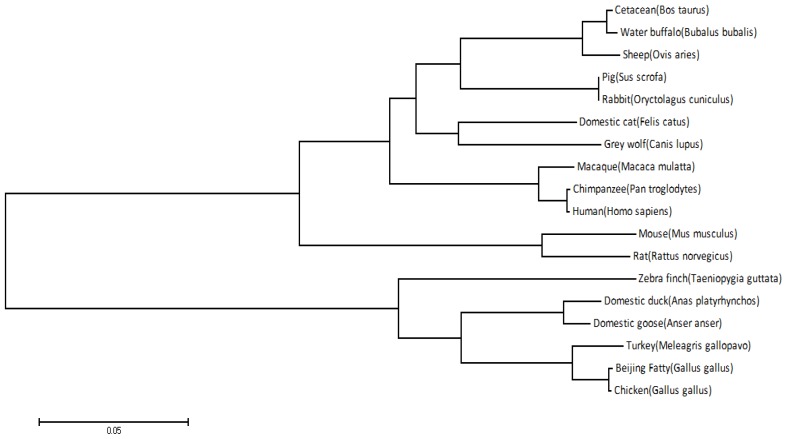
Phylogenetic analysis of *duck TLR4*. The neighbor-joining tree was constructed using MEGA5.2. The sequences were derived from the predicted amino acid sequences of the Jinding duck *TLR4* (AEL97644) and the following GenBank entries for duck (accession number: JN048668), chicken (*Gallus gallus*, accession number: NM_001030693), goose (accession number: HQ436371), turkey (accession number: XM_003211211), zebra finch (accession number: NM_001142454), and Beijing Fatty chicken (accession number: JQ711152). The scale bar is 0.05. The unrooted tree was generated using ClustalX program by neighbour-joining method. Bootstrap values were derived from 1000 replicate runs.

An alignment of TLR4 amino acid sequences of the above species is shown in Figure 2. A phylogenetic tree for *TLR4* based on 18 species is shown in Figure 3.

The duck *TLR4* sequence shows the highest sequence homology with goose *TLR4* (96.8%, Figure 3). Homology between chicken, turkey, and red jungle fowl sequences is also high, but the sequence homology between zebra finches and the other species is lower. As shown in Figure 3, Beijing fatty chicken and red jungle fowl are tightly clustered into one class, and then further clustered with turkey, duck, and goose, and more distantly with zebra finch. This phylogenetic tree is consistent with the genetic relationship of those species. Thus, the *TLR4* gene is conserved within the evolutionary process.

### 2.2. Tissue Distribution of *Duck TLR4* mRNA

**Figure 4:**
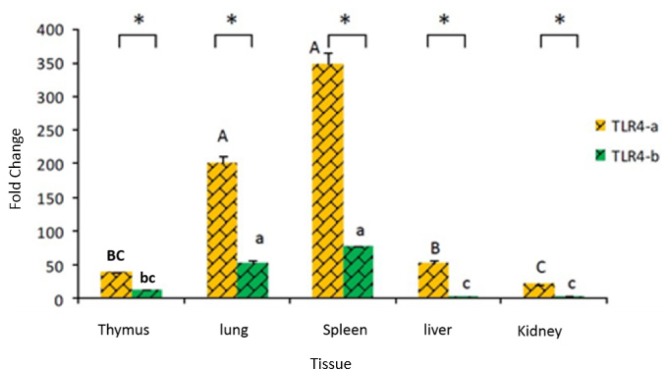
Tissue distribution of duck *TLR4* mRNA expression. *Y*-axis indicates fold change of the expression, *X*-axis indicates different tissues of duck; “*” indicates *p* < 0.05. The difference between bars with the same letter is not significant, the difference between adjacent capital letters (A, B, C) (*p* < 0.05) and small letters (a, b, c) (*p* < 0.01) represent a significant difference. The relative mRNA levels of individual *TLR4-a* and *TLR4-b* genes normalized with respect to housekeeping gene *GAPDH*.

In healthy tissue, the highest expression of *TLR4-a* was seen in the spleen and lung. The expression of wild-type *TLR4-a* was significantly higher than that of the variant form *TLR4-b* (*p* < 0.05) in all tissues tested and especially in the liver and kidney (Figure 4).

### 2.3. Expression of *TLR4-a* and *TLR4-b* Following Stimulation with LPS

**Figure 5:**
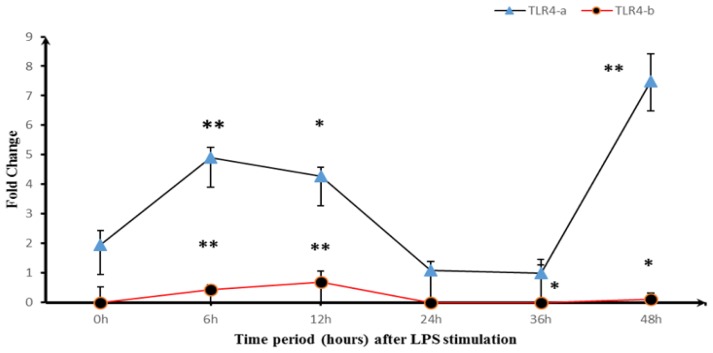
*TLR4-a* and *TLR4-b* mRNA levels in LPS-stimulated DEF cell cultures. *Y*-axis indicates fold change of the expression, *X*-axis indicates time period (h) after LPS stimulation; DEF cells were collected 0 h, 6 h, 12 h, 24 h, 36 h, and 48 h after stimulation. RNA was extracted and reverse transcribed, and the expression level of the two alternative splice isoforms, *TLR4-a* and *TLR4-b*, was measured. The relative mRNA levels of individual *TLR4-a* and *TLR4-b* genes normalized with respect to housekeeping gene *GAPDH*. “*” indicates *p* < 0.05, and “**” indicates *p* < 0.01.

Normal duck embryo fibroblasts (DEFs) were stimulated with LPS and cells were collected at 0 h, 6 h, 12 h, 24 h, 36 h, and 48 h. RNA was extracted and reverse transcribed, and the expression level of the two alternative splicing isoforms was compared (Figure 5). DEFs stimulated with LPS showed enhanced expression of both *TLR4-a* and *TLR4-b*, reaching a peak at 6 h and 12 h, followed by a sharp decline over time. Expression of *TLR4-a* subsequently increased again at 36 h, whereas expression of *TLR4-b* remained very low.

DEFs were transiently transfected with a pcDNA3.1(+)-*TLR4* expression vector and stimulated with LPS or Poly(I:C) (a dsRNA mimetic) to simulate infection with Gram-negative bacteria or viruses, respectively.

**Figure 6:**
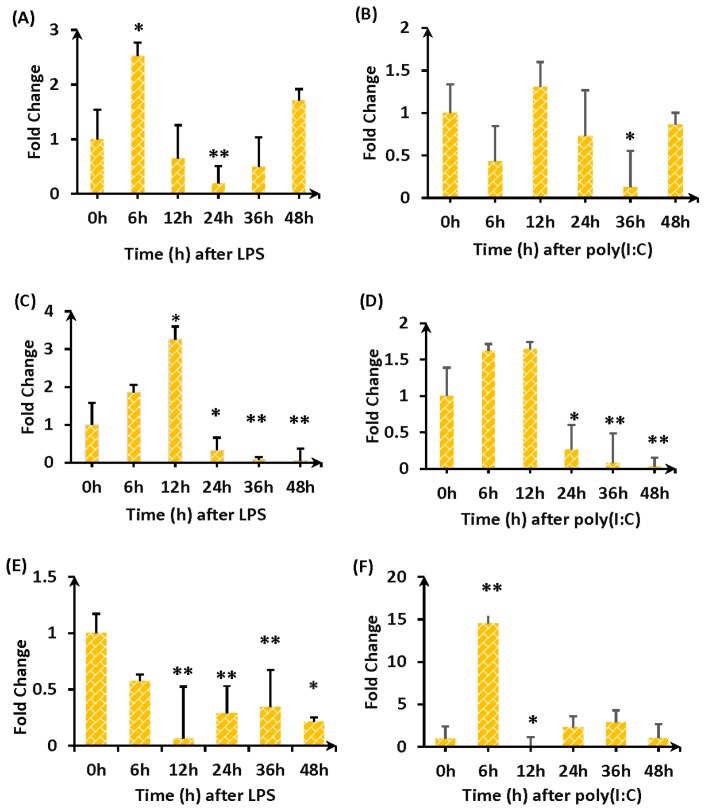
Changes in mRNA expression following stimulation of transfected DEFs with LPS or Poly(I:C). *Y*-axis indicates fold change of the expression, *X*-axis indicates time period (h) after LPS stimulation or Time period (h) after poly (I:C) stimulation; stimulants (LPS or Poly(I:C)) and genes (*IL-1*β (**A** and **B**), *IL-6* (**C** and **D**), and *MHC II* (**E** and **F**)) are indicated below each figure. Cells were harvested at the times indicated, RNA was prepared, and RT-PCR was performed with *GAPDH* as the internal control. Significant differences in expression compared with the untreated control (0 h) were calculated using two-directional paired Student’s *t*-test. *P*-values < 0.05 were considered to be statistically significant, and are indicated as “*” for *p* < 0.05 *vs.* 0 h, “**” for *p* < 0.01 *vs.* 0 h.

After stimulation with LPS or poly(I:C), cells were collected and RNA was extracted at 0 h, 6 h, 12 h, 24 h, 36 h, and 48 h. Quantitative fluorescence was used to detect expression changes in genes encoding the immune modulators *IL-1*β (Figure 6A,B), *IL-6* (Figure 6C,D), and *MHC II* (Figure 6E,F). Either LPS or poly (I:C) stimulation induced *TLR4* gene overexpression in fibroblasts, *IL-1*β expression initially increased and then decreased, followed by a second increase in LPS-stimulated cells only. *IL-6* expression showed a sharp increase in both LPS- or poly (I:C)-stimulated samples within 12 h, and then showed a sharp decline in expression. *MHC II* expression increased within 12 h after poly(I:C) stimulation and then decreased (12–48 h).

## 3. Discussion

TLR 4 is a specific receptor that can detect LPS present in most Gram-negative bacteria and regulate the signal transduction events induced by LPS, and therefore plays an important role in pathogen recognition and activation of the innate immune system [11]. TLR4 has been shown to play a significant role in the susceptibility of mammals and chickens to systemic salmonellosis [12].

Bacterial infections cause worldwide diseases each year [13], dramatically influencing the performance of livestock. The TLR4-mediated immune response is the first line of defense against bacterial infection. Several earlier studies have addressed expression of the duck *TLR4* gene and protein during pathogenic challenge [4]. In most of these studies, activation of *TLR4* was monitored by measuring the level of a transcript harboring the third exon of a previously reported transcript variant. A recent study has shown that more than 40% of human gene transcripts undergo alternative splicing, notably many of the genes involved in immunological responses [14,15]. Alternatively spliced proteins probably function in regulation of the immune response. This study focused on *TLR4* splice variants in the duck and their function.

We identified a splice variant in duck, *TLR4-b*, which retains the first intron of 835 bp in length. There are two mechanisms by which this may affect the translation process: (A) introduction of a premature termination codon at 291 bp, leading to translation of only 76 amino acid residues; or (B) a reading frame shift (RFS) that starts translation from 967 bp, resulting in a 76-amino acid deletion and leading to removal of the first transmembrane domain. Previous studies have reported four alternative splice isoforms in humans [16], two in mice [17], three in sheep [7], and three in pigs [18]. Whether there are additional alternatively spliced forms in the duck requires further investigation. These findings strongly suggest that alternative TLR4 splice isoforms have a large impact on TLR4 function.

In our experiment, we found a constitutive expression of *TLR4* gene in various tissues. Besides, we also detected that *TLR4-a* mRNA was significantly increased after LPS challenge, while a slight change of *TLR4-b* was observed. It is evident from these findings that duck *TLR4*’s function is dependent on *TLR4-a*.

A reliable method was developed to monitor *TLR4* transcript variants individually after LPS stimulation of duck fibroblasts. Total *TLR4* expression was rapidly down-regulated within the first 6 h following LPS stimulation, subsequently increased (8–12 h) and declined thereafter. It has been shown previously that expression of TNF-α, a marker of LPS-derived activation, was dramatically elevated within 0.5 h, indicating that LPS-induced immune responses are immediately triggered [19]. It is known that *TLR4* shows distinct patterns of expression in different type of cells. For example, the degradation of *TLR4* observed in monocytes is associated with LPS tolerance, helping the animal survive bacterial infection [20]. It is notable that fibroblasts are a kind of antigen-presenting cell that can trigger immunological responses through the release of large amounts of pro-inflammatory cytokines. Because fibroblasts are the most common cell type within the body, it is reasonable to assume that *TLR4* expression in fibroblasts is tightly regulated.

The expression of *TLR4-a* was consistent with that of total *TLR4*, indicating that *TLR4-a* is the functional transcript variant. It is likely that these transcript variants have biological relevance because alternative splicing is a powerful and efficient regulatory mechanism known to regulate gene expression and protein production [21]. It has frequently been observed that the immune system regulates its own response during pathogen infection.

Emerging results indicate that *TLR4-a* is an important immune-related gene in the duck, in part because it is expressed preferentially in immune or immune-related organs under normal conditions. The duck *TLR4* gene shows the same organization as *TLR4* genes from sheep, mouse, chicken, goose, and pig, with three exons and two introns, demonstrating that this gene has been conserved during evolution. Conserved structural features are also present within the duck homologs. Mutations in *TLR4* have been associated with differences in LPS responsiveness. According to the current model, *TLR4* interacts with three different extracellular proteins: LPS binding protein (LBP), CD14, and myeloid differentiation protein 2 (MD-2), leading to LPS-mediated NF-κB activation and the production of proinflammatory cytokines [22,23]. Thus, *TLR4* plays an important role in the defense against infections by Gram-negative bacteria that release LPS.

In this study, we used LPS to stimulate duck embryo fibroblasts and monitored expression of the effector genes *IL1*β, *IL6*, and *MHC II* in the *TLR4* pathway [24]. Expression of these genes generally showed an upward trend immediately after stimulation, followed by a decrease and then a second increase. TLR4 is part of a complex immune system network and plays a key role in protection as an important regulator of components present in many infectious diseases. Sheng *et al.* found that LPS-induced expression of *TLR4* is rapidly elevated and maintained at a high level during the entire *TLR4* process [25,26]. In T lymphocytes, thymus, and spleen tissue macrophages, LPS can significantly increase expression of *TLR4* without affecting gene expression. In human polymorphonuclear leukocytes and monocytes, *TLR4* expression is significantly increased upon stimulation.

Cell and tissue distribution are important determinants of *TLR4* function, as they influence the capacity to detect different microorganisms during their entry and growth in different tissues. Understanding the expression patterns of duck TLRs will enable a better interpretation of immune induction and the host-pathogen relationships that define infectious disease biology in the duck [16,27]. A number of studies have defined the response of cultured chicken cells after exposure to a variety of challenges with microorganisms or their products [28]. In the present study, *duTLR4* mRNA was expressed extensively in all normal tissues examined, including the liver, kidney, spleen, thymus, and lung. These data were consistent with those for mammalian and chicken *TLR4*. In mammals, fatty acids are able to activate *TLR4*, resulting in enhanced secretion of proinflammatory chemokines and cytokines such as tumor necrosis factor-α and IL-6. Mice deficient in *TLR4* are protected from obesity-induced insulin resistance and inflammatory insults. Additionally, TLR4 is a molecular link between nutrition, lipids, and innate immunity. The extensive distribution of functional TLR4 in various duck tissues may contribute to a variety of immune responses.

## 4. Experimental Section

### 4.1. Animals and Tissue Sample Collection

Jinding ducks (*Anas platyrhynchos*; 3 days old) were obtained from the National Waterfowl Germplasm Resource Gene Pool (Taizhou, China). The ducks (*n* = 80) were slaughtered by exsanguination according to protocols approved by the Animal Care Advisory Committee of Yangzhou University and blood samples were collected for DNA extraction. The liver, lung, spleen, kidney, and thymus were removed and immediately frozen in liquid nitrogen before storage at −80 °C for RNA isolation.

### 4.2. DNA and RNA Isolation and cDNA Synthesis

Genomic DNA was isolated using the TIANamp Genomic DNA Kit according to the manufacturer’s instructions (Tiangen Biotech (Beijing) Co., Ltd., Beijing, China). Total RNA was extracted from collected tissue samples or cells using RNAiso Plus (TaKaRa, Dalian, China) according to the manufacturer’s instructions. Total RNA concentrations were determined using a Beckman Coulter DU800 spectrophotometer (THERMO, Fullerton, CA, USA). Reverse transcription was performed using TransScript First-Strand cDNA Synthesis SuperMix (Tiangen Biotech (Beijing) Co., Ltd., Beijing, China).

### 4.3. Cloning of the *TLR4* Gene

The DNA and mRNA sequences of *TLR4* (GenBank ID: JN048668) were amplified using the primers listed in Table 1. The polymerase chain reaction (PCR) was performed in 20-μL reactions containing 0.4 μL of first-strand cDNA, 1 μL each of 10 μM forward and reverse primers, 7.6 μL DEPC-treated water, and 10 μL of 2 × Taq PCR MasterMix (Tiangen, Beijing, China). Thermal cycling conditions were as follows: 1 cycle of 95 °C for 5 min, then 32 cycles of 94 °C for 30 s, 65 °C for 30 s, and 72 °C for 2 min 30 s, followed by 1 cycle of 72 °C for 10 min. PCR products were analyzed by electrophoresis on 1.0% agarose gels, purified using a gel extraction kit (Dongsheng Biotech Co., Ltd., Beijing, China), ligated into the pMD19-T Vector (TaKaRa, Dalian, China), transformed into *E. coli* DH5α cells, and sequenced.

### 4.4. Construction of pcDNA 3.1(+)-*TLR4* Overexpression Vector

A DNA fragment containing the entire open reading frame of duck *TLR4* was PCR amplified using the specific primers pcDNA3.1(+)-*TLR4*-F (5′-ggaattcCATATGATGCCCAGGAGAGCAGCTCTCCTC-3′); and pcDNA3.1(+)-*TLR4*-R (5′-gcTCTAGACATGAGCTTTTCCTCCTCGTGATTCC-3′) and the plasmid pMD19-T-*TLR4* as a template. Primer pcDNA3.1(+)-TLR4-F introduced a *Nde* I site, and primer pcDNA3.1(+)-TLR4-R introduced a *Xba* I site (underlined). After digestion with *Nde* I and *Xba* I, the PCR product was inserted into the vector pcDNA3.1(+) (Invitrogen, Carlsbad, CA, USA). Proper plasmid construction was confirmed by sequencing.

### 4.5. Sequence Analysis, Multiple Sequence Alignment, and Phylogenetic Analysis

Searches for nucleotide and protein sequence similarities were conducted with the BLAST algorithm from the National Center for Biotechnology Information (NCBI, http://blast.ncbi.nlm.nih.gov/Blast.cgi) [29]. Based on the duck *TLR4* sequence (GenBank ID: JN048668), primers pMD19-T-TLR4-F and pMD19-T-TLR4-R were designed and used to amplify the potential *TLR4* cDNA sequence from total RNA extracted from the liver of a Jinding duck. The deduced amino acid sequence of TLR4 was analyzed with the Expert Protein Analysis System (Expasy, http://www.mrc-lmb.cam.ac.uk/genomes/madanm/pres/swiss1.htm) [30]. Multiple sequence alignment of *TLR4* CDS and DNA was performed with the ClustalW multiple alignment program (http://www.ebi.ac.uk/Tools/clustalw2/) and the Multiple Align Show program (http://www.bioinformatics.org/sms/). Using a neighbor-joining (NJ) algorithm, an unrooted phylogenetic tree was constructed based on the deduced amino acid sequences of TLR4 using MEGA 5.05 software (http://www.megasoftware.net). To derive confidence values for tree nodes, bootstrap values were obtained for 1000 replications [31–33].

A phylogenetic tree was constructed from the *TLR4* coding regions of 18 different species: the domestic duck (*Anas platyrhynchos*, accession number: JN048668), red jungle fowl (*Gallus gallus*, accession number: NM_001030693), the domestic goose (*Anser anser*, accession number: HQ436371), chicken (*Gallus gallus domesticus*, accession number: JQ711152), cattle (*Bos Taurus*, accession number: NM_174198.6), chimpanzee (*Pan troglodytes*, accession number: NM_001144863.1), cat (*Felis catus*, accession number: NM_001009223.1), gray wolf (*Canis lupus*, accession number: NM_001002950.2), human (*Homo sapiens*, accession number: NM_138554.4), macaque (*Macaca mulatta*, accession number: NM_001037092.1), mouse (*Mus musculus*, accession number: JX878359.1), pig (*Sus scrofa*, accession number: NM_001113039.1), rabbit (*Oryctolagus cuniculus*, accession number: NM_001113039.1), rat (*Rattus norvegicus*, accession number: NM_019178.1), sheep (*Ovis aries*, accession number: NM_001135930.1), wild turkey (*Meleagris gallopavo*, accession number: XM_003211211.1), water buffalo (*Bubalus bubalis*, accession number: HQ343416.1), and zebra finch (*Taeniopygia guttata*, accession number: NM_001142454.1).

### 4.6. Real-Time Quantitative PCR

Real-time quantitative PCR was performed using the Applied Biosystems 7500 Real-Time PCR Detection System (Applied Biosystems, Foster City, CA, USA). The gene-specific primers used are listed in Table 1. Each PCR mixture contained 2 μL first-strand cDNA, 0.4 μL each of 10 μM forward and reverse primers, 6 μL DEPC-treated water, and 10 μL of 2 × SYBR qPCR Mix (TaKaRa, Dalian, China). The PCR cycling conditions were as follows: 95 °C for 10 s, then 40 cycles of 95 °C for 15 s and 60 °C for 30 s. Each primer pair yielded a single peak in the melting curve and a single band of the expected size on an agarose gel. The identities of the PCR products were confirmed by sequencing. The *duTLR4* and *GAPDH* genes were detected simultaneously at least three times. Data were analyzed according to the efficiency-corrected comparative Ct method and normalized using *GAPDH* expression levels.

### 4.7. Expression of *TLR4* in Different Tissues

To study the tissue distribution of *duTLR4-a* and *duTLR4-b* expression, we performed RT-PCR on total RNA isolated from the thymus, liver, spleen, lung, and kidney of healthy ducks. Amplification was performed using the following cycling parameters: 30 cycles of 94 °C for 30 s, 60 °C for 30 s, and 72 °C for 30 s. Primers are shown in Table 1. After the reaction, PCR products were visualized on 1.2% agarose gels. Expression levels were assessed by comparison with an internal standard, duck glyceraldehyde-3-phosphate dehydrogenase (*GAPDH*).

### 4.8. *duTLR4* Expression in Duck Embryo Cells after LPS and Poly (I:C) Challenge

The DEF cell line was originally derived from a duck embryo (ATCC, Manassas, VA, USA). DEF cells were maintained in Dulbecco’s modified Eagle medium (DMEM) medium (Invitrogen, Carlsbad, CA, USA) supplemented with 10% fetal bovine serum (FBS) (Invitrogen, Carlsbad, CA, USA) at 37 °C, 5% CO_2_ in a humidified atmosphere. Medium was renewed every 3 days. DEF cells were seeded in 24 well plates (Corning, Corning, NY, USA) at a density of 2.0 × 10^5^ cells/well 24 h prior to transfection. Cells were transfected with 2 μg of the plasmid pcDNA3.1(+)-*TLR4-a* or an equal amount of the empty vector pcDNA3.1(+) using Lipofectamine 2000 (Invitrogen, Carlsbad, CA, USA). Transfected DEF cells (0.5 mL) were transferred to each well of a 24-well cell-culture dish containing 1.5 mL supplemented DMEM (Invitrogen) (10% FBS) and 50 μg/mL Poly(I:C) (Sigma, St. Louis, MO, USA), 1 μg/mL LPS (Sigma, *E. coli* 0127:B8), or PBS as a control. Cells were collected after 0 h, 6 h, 12 h, 24 h, 36 h, and 48 h incubation at 37 °C with 5% CO_2_. The samples assayed at the 0 h time point were harvested immediately after mixing with the supplemented DMEM. RNA was extracted and subjected to qRT-PCR.

### 4.9. Statistical Analysis

Data are expressed as mean ± standard deviation (SD). Student’s *t*-tests were used to examine differences in expression, with a *p*-value < 0.05 indicating statistical significance. RT-PCR data were expressed as mean ± SD. One-way ANOVA and Tukey’s tests (V22.0; SPSS Inc., Chicago, IL, USA, 2013) were performed to assess the significance of between-treatment differences, with a *p*-value < 0.05 indicating statistical significance.

## 5. Conclusions

Stimulation of DEF cells overexpressing the *TLR4* gene with LPS or poly(I:C) induced expression of *IL-1*β, *IL-6*, and *MHC II*. Moreover, *in vivo* levels of *TLR4* expression were increased in the thymus, liver, spleen, lung, kidney, and other tissues after stimulation. These results indicate that duTLR4 plays an important role in both the body’s resistance to bacterial infections and the anti-virus response.

## Figures and Tables

**Table 1:** Primers used for gene cloning, mapping, and expression analysis.

Primer name	Sequence (5′→3′)	*T*m (°C)	Note
*P1F*	CACTTCCCCTTGTTCTCTGC	65	coding sequence amplification
*P1R*	AGAGGCAGACAAATGGATGG		
*IL-1*β*-F*	CCATGGTGCTTTCTCCTGTGT	60	RT-qPCR
*IL-1*β*-R*	GAGCTTGTAGCCCTTGATGC		
*IL-6-F*	GAGCTTGTAGCCCTTGATGC	60	RT-qPCR
*IL-6-R*	TTCGACGAGGAGAAATGCTT		
*MHC II-F*	CCACCTTTACCAGCTTCGAG	60	RT-qPCR
*MHC II-R*	CCGTTCTTCATCCAGGTGAT		
*P3F*	CCATGGTGCTTTCTCCTGTGT	60	RT-qPCR
*P3R*	CTGGGTGGTGTTTGGGACTT		
*P4F*	CCCTCTGCTTGGGAGATTTG	60	RT-qPCR
*P4R*	GCTTGTTCTGTTTCTCAGGTGTTT		
*GAPDH-F*	TGCTAAGCGTGTCATCATCT	60	RT-qPCR
*GAPDH-R*	AGTGGTCATAAGACCCTCCA		
